# Differential Palmit(e)oylation of Wnt1 on C93 and S224 Residues Has Overlapping and Distinct Consequences

**DOI:** 10.1371/journal.pone.0026636

**Published:** 2011-10-26

**Authors:** Lisa M. Galli, Laura W. Burrus

**Affiliations:** Department of Biology, San Francisco State University, San Francisco, California, United States of America; University of Oldenburg, Germany

## Abstract

Though the mechanisms by which cytosolic/intracellular proteins are regulated by the post-translational addition of palmitate adducts is well understood, little is known about how this lipid modification affects secreted ligands, such as Wnts. Here we use mutational analysis to show that differential modification of the two known palmit(e)oylated residues of Wnt1, C93 and S224, has both overlapping and distinct consequences. Though the relative roles of each residue are similar with respect to stability and secretion, two distinct biological assays in L cells show that modification of C93 primarily modulates signaling via a ß-catenin independent pathway while S224 is crucial for ß-catenin dependent signaling. In addition, pharmacological inhibition of Porcupine (Porcn), an upstream regulator of Wnt, by IWP1, specifically inhibited ß-catenin dependent signaling. Consistent with these observations, mapping of amino acids in peptide domains containing C93 and S224 demonstrate that acylation of C93 is likely to be Porcn-independent while that of S224 is Porcn-dependent. Cumulatively, our data strongly suggest that C93 and S224 are modified by distinct enzymes and that the differential modification of these sites has the potential to influence Wnt signaling pathway choice.

## Introduction

Proteins often undergo post-translational modifications that are critical for their function. The covalent attachment of fatty acids (acylation) is one such modification. Proteins can be modified by fatty acids of different chain lengths; palmitoylation is the addition of a 16 carbon fatty acid. Palmitate can be linked to amino groups (N-linked), sulfhydryl groups (S-linked) or alcohol groups (O-linked). S-Palmitoylation of cytosolic proteins and the intracellular domains of transmembrane proteins is known to control membrane association, targeting to lipid rafts and intracellular membranes, intracellular trafficking, protein-protein interactions, biological activity and stability [1], [2], [3], [4], [5].

More recently, the N-,O- and S-palmitoylation of “secreted” ligands such as Hedgehog, Spitz and Wnt has also been reported to play roles in regulating the activity and distribution of these proteins [6], [7], [8], [9], [10], [11], [12], [13], [14], [15]. Despite numerous advances, however, the functional role(s) of lipid modifications to “secreted” proteins remains poorly understood.

Mass spectrometry studies have definitively demonstrated the acylation of Wnt3a with two lipid adducts, fully saturated palmitate (C16:0) on a conserved cysteine (C77; S-palmitoylation) and mono-unsaturated palmitoleic acid (C16:1) on a conserved serine (S209; O-palmiteoylation) [13], [14]. These residues are invariant amongst all of the 19 vertebrate Wnt family members and all, but one, of the known invertebrate Wnt family members [16]. Additional mass spectrometry studies have confirmed the palmitoylation of the cysteine residue in Wnt5a [10]. Thus, it seems likely that the acylation of these residues is conserved across family members.

As the precise regulation of Wnt signaling is required for proper embryonic development and adult homeostasis [17], [18], [19], [20], [21], [22], it is crucial to fully understand the functional roles of the lipid modifications to the cysteine and serine residues. To achieve this goal, we tested a panel of Wnt1 and Wnt3a constructs encoding differentially acylated Wnt proteins for stability, secretion, and biological activity. We found that mutation of either the cysteine or the serine has similar effects on stability and secretion, but that the relative importance of each residue for biological activity in ß-catenin dependent and independent assays differs significantly.

Of equal importance is the identification of the upstream regulators of these modifications. Porcupine (Porcn) and Wntless (Wls) are upstream regulators of Wnt signaling. While Porcn is predicted to play a role in Wnt palmit(e)oylation [13], [23], Wls is thought to escort palmit(e)oylated Wnts through the secretory pathway [24], [25], [26], [27]. Though it has not been experimentally demonstrated that Porcn acts directly to palmit(e)oylate Wnts, bioinformatic studies have identified it as a putative O-acyl transferase [23]. Additional studies show that Porcn is required for the palmiteoylation of Wnt3a S209 [13]; however, it is not known if Porcupine participates in the palmitoylation of the cysteine residue [28]. Our studies in L cells show that pharmacological inhibition of Porcn significantly reduces Wnt1 signaling via the ß-catenin dependent pathway, but not a ß-catenin independent pathway (that is yet to be defined). To determine whether Porcn is likely to be involved in the palmit(e)oylation of one or both lipid modified sites, we tested the ability of Porcn to modify GFP-tagged Wnt1 peptides targeted to the secretory pathway. Specifically, we used a hydrophobicity assay along with an assay for co-localization with lipid rafts to identify Wnt1 peptides with sites for Porcn-dependent modifications. Our data from these studies are consistent with a model in which the cysteine residue is modified in a Porcn-independent manner while the serine residue is modified in a Porcn-dependent manner.

In sum, our results strongly suggest that distinct enzymes are involved in the lipid modification of the cysteine and serine residues and that Porcn is specifically involved in the palmiteoylation of the serine residue. We also provide evidence for the existence of at least one additional site for Porcn-dependent lipid modification. Our results further indicate that differential regulation of the lipid-modification of the cysteine and serine residues may influence the “choice” of Wnt signaling pathways.

## Results

### Monoclonal antibody against chick Wnt1 recognizes palmit(e)oyl-modified Wnt1

**Figure 1:**
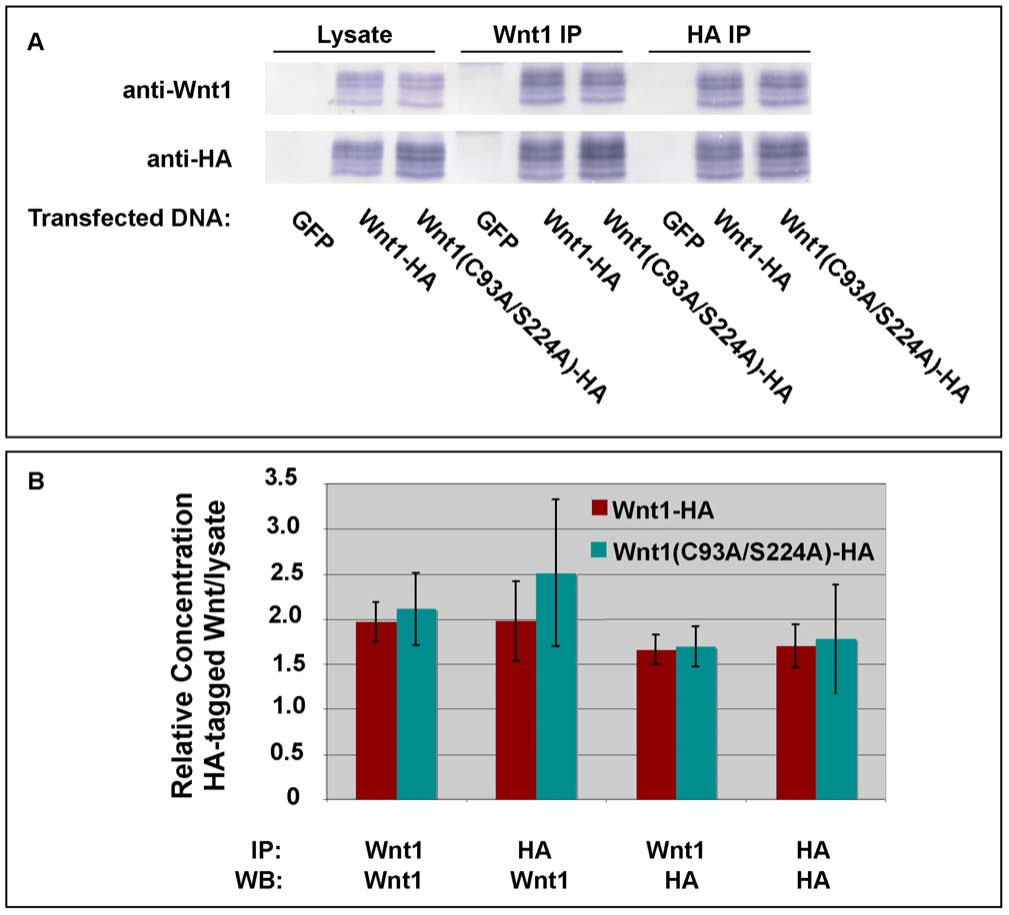
Wnt1 monoclonal antibody recognizes wild-type and lipid-deficient Wnt1 proteins. HEK293T cells were transiently transfected with constructs encoding either GFP (control), Wnt1-HA or Wnt1 C93A/S224A-HA. Cell lysates were immunoprecipitated with anti-HA or anti-Wnt1 (MAb 7B3-A10-F9), separated by SDS-PAGE and blotted onto PVDF. Western Blots were then probed with anti-HA or anti-Wnt1 (MAb 5F1-G11-D1) as indicated (**Panel A**). Blots were scanned and analyzed using NIH ImageJ. The quantitative results are shown in the graph in **Panel B**. This experiment was performed twice. Error bars indicate the standard error.

We have previously reported the development of monoclonal antibodies against Wnt1 [7]. Because the antibodies were raised against a peptide that spans one of the known palmitoylation sites of Wnt1 (C93), we sought to ensure that the antibody used for subsequent studies recognizes lipid-modified and unmodified Wnt1 equally well (MAb 7B3-A10-F9). To do this, we performed immunoprecipitation experiments with HA-tagged Wnt1 and HA-tagged Wnt1 C93A/S224A (lacking the two known lipid modification sites), using different combinations of HA and Wnt1 antibodies for the immunoprecipitations and Western blots (Fig. 1). Quantitation of these data shows that the Wnt1 antibody works equally well on wild-type Wnt1 and Wnt1 lacking both of the known palmit(e)oylation sites (Fig. 1B). These data are in concordance with preliminary data showing that Wnt1 MAb 7B3-A10-F9 can immuoprecipitate Wnt1 that has been radiolabeled with ^3^H-palmitate (data not shown).

### The lipid modification of Wnt1 is required for Wnt secretion, but not stability

Though conserved cysteine and serine residues have been shown to be lipid modified in vertebrate Wnts, no comprehensive quantitative analysis of the relative roles of these two sites in the secretion and stability of vertebrate Wnts has been reported. Thus, we sought to test how lipid modifications of the cysteine and serine residues affect Wnt1 stability and secretion. Because Wnts are notoriously poorly secreted from most cell lines, including HEK293T cells, we performed these pulse-chase studies in stably transfected murine L cells, which secrete Wnt proteins far more efficiently than other vertebrate cell lines [29]. Like HEK293T cells, L cells express low levels of endogenous Porcn (data not shown).

**Figure 2:**
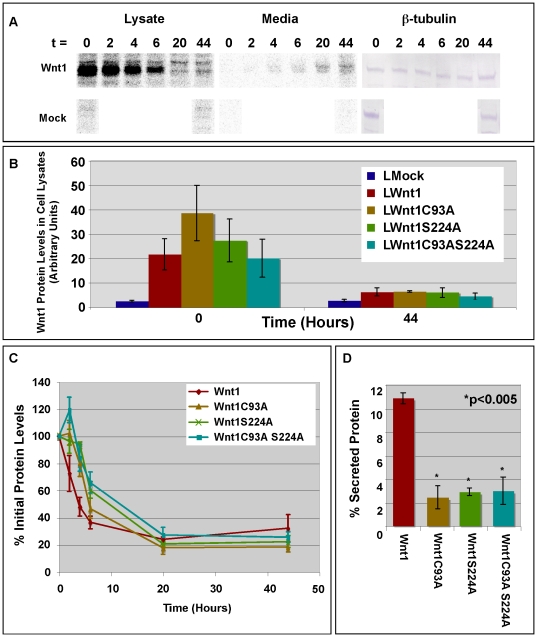
Pulse-chase analysis of Wnt1 stability and secretion. **Panel A:** Stably transfected L cells were pulsed with ^35^S-cys and ^35^S-met for 4 hours prior to chasing for 0–44 hours with unlabeled media. Wnt1 was immunoprecipitated from cell lysates and media using anti-Wnt1 monoclonal antibodies developed in our lab [7]. Immunoprecipitated protein was separated by SDS-PAGE and subjected to analysis with a Kodak STORM Phosphorimager. A representative scan for wild-type Wnt1 is shown. As a control, a Western blot of total protein in the cell lysates prior to immunoprecipitations was also performed using antibodies against ß-tubulin. **Panel B:** This graph shows quantitation of the radiolabel in each sample (lysate only) as determined by Phosphorimaging. Data for mock transfected L cells and Wnt1 (wild-type and mutant) transfected L cells is shown for the 0 and 44 hr time points. These data were not normalized in any way. Data from 2–3 independent replicates is shown; error bars show standard error. **Panel C:** This graph shows quantitation of the total radiolabel in each sample (lysate+medium) as determined by Phosphorimaging. Identical exposures were used for cell lysates and media. Values for time = 0 are set to 100%. Data from 2 or 3 independent replicates is shown; error bars show the standard error. **Panel D:** This graph shows the percent of secreted Wnt1 protein (Medium_max_/Lysate_max_*100). Results from 2–3 independent replicates are shown. Error bars show standard error. The percentage of secreted Wnt1C93A, Wnt1S224A, and Wnt1C93AS224A are all significantly less than that of wild-type Wnt1 (p<0.005). However, there are no statistically significant differences between the various mutant Wnt1 proteins.

To quantitatively assess the movement of wild-type and mutant Wnt1 through cells into the media, we pulsed cells with ^35^S-labeled cysteine and methionine for 4 hrs and then chased with unlabeled medium for the specified time intervals (Fig. 2). Wnt1 was then immunoprecipitated from cells and media, separated by SDS-PAGE, and subjected to quantitative analysis with a Kodak Phosphorimager, using identical exposure times for all samples (Fig. 2). A representative scan is shown for wild-type Wnt1 in Fig. 2A. Scans for wild-type and mutant Wnt1 were quantitated for all time points. Importantly, our data show that at the start of the chase (t = 0), each of the L cell clones chosen for analysis showed roughly equal levels of wild-type and mutant Wnt1 (Fig. 2B). Though Wnt1 C93A showed slightly higher levels than wild-type Wnt1, Wnt1 S224A, and Wnt1 C93AS224A (Fig. 2B), these differences were not statistically significant. As expected, the levels of wild-type and mutant Wnt1 in cell lysates were dramatically reduced after 44 hrs of chase (Fig. 2B).

To examine the kinetics of Wnt1 turnover, we then compared the stability of wild-type and mutant Wnt1 proteins by plotting the total amount of ^35^S-labeled Wnt protein found in cell lysates and media at each time point (Fig. 2C). To facilitate comparisons, the total amount of ^35^S-labeled Wnt1 protein (wild-type and mutant) at each time point was normalized to the total amount present at the beginning of the chase (t = 0 set to 100%). By calculating the half-life of the protein using data obtained between 2 and 6 hrs, we found that the half-life of wild-type Wnt1 is 4.4+/−0.7 hrs (n = 3) while that of the Wnt1 double mutant is 5.3+/−1.5 hrs (n = 3). As these numbers are not significantly different in a Student's t-test (P = 0.6), our studies do not indicate a clear role for Wnt lipidation as a regulator of stability.

Interestingly, wild-type Wnt1 shows a classic logarithmic decay while all of the mutant Wnt1 variants show somewhat unusual decay patterns (Fig. 2C). For instance, Wnt1 (C93A) and Wnt1 (C93AS224A) show extremely reproducible increases in the amount of radioactivity after 2 hrs of chase as compared to 0 hrs. Similarly, Wnt1 S224A levels decline less than expected over the first 4 hrs. While we do not know the reason for these unusual kinetics, we suspect these data reflect alterations in the rate of Wnt folding. For instance, if mutation of Wnt1 slows folding, the delay might inhibit our ability to immunoprecipitate Wnt protein with antibodies that are possibly conformation specific.

We then compared the secretion of wild-type and acylation deficient Wnt1 variants (Fig. 2D). To calculate the percent of secreted Wnt protein, we divided the maximum amount of Wnt1 in the media (generally at 44 hrs) by the maximum amount of Wnt1 in the cells (at either 0 or 2 hrs). We found that 10.5+/−0.4% of wild-type protein was secreted. By contrast, the secretion of the single mutants and the double mutant was reduced 3 to 4 fold. The similar level of impairment for the C93A and S224A mutants suggests an equal requirement for both C93 and S224 in Wnt1 secretion from L cells (Fig. 2D). Though the double mutant appears to be secreted at slightly higher levels than the single mutants, comparison of the double mutant to the single mutant shows no significant differences and leads us to conclude that the secretion of the double mutant is similar to that of the single mutants (Fig. 2D). In sum, our results lead us to conclude that both the cysteine and serine residues are required for the secretion of Wnt1 from L cells.

### The lipid-modified cysteine and serine residues are not required for the release of Wnt1 from the cell surface

**Figure 3:**
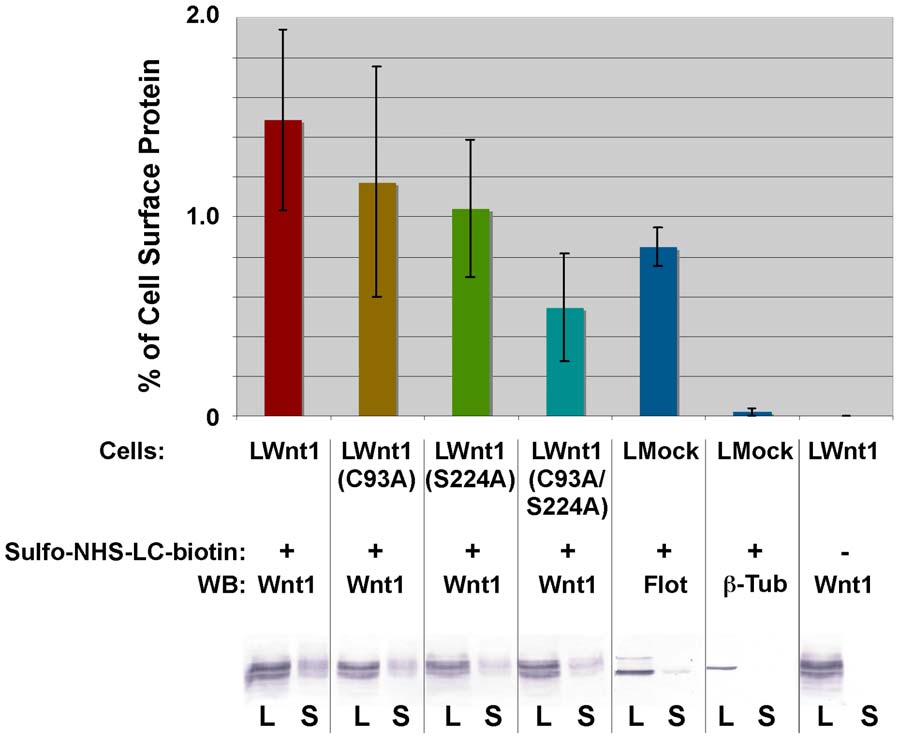
Lipid-deficient Wnt1 does not accumulate on the surface of L cells. L cells stably transfected with wild-type and mutant Wnt1 constructs were treated with 100 ug/ml of heparin to release Wnt1 protein bound to HSPGs. After labeling cells with Sulfo-NHS-LC-biotin, cell surface proteins (S) were immunoprecipitated using NeutrAvidin beads. Aliquots of the initial lysates (L; before immunoprecipation) and the immunoprecipitated material were subjected to SDS-PAGE, blotted onto PVDF, and probed with the indicated antibodies. We used Flotillin (cell surface) and ß-tubulin (intracellular) as internal controls. Blots were scanned and quantitated using NIH ImageJ. This experiment was replicated 6 times. Error bars represent the standard error.

The observed defects in secretion could reflect poor release of lipid-deficient Wnts from the cell surface or the inefficient transit of lipid-deficient Wnt proteins through the secretory pathway. To compare the levels of wild-type and mutant Wnt1 localized to the cell surface, we labeled all exposed primary amino groups on intact L cells with Sulfo-NHS-LC-biotin. After quenching the reaction, Wnt1 present on the cell surface was detected by immunoprecipiation with NeutrAvidin followed by SDS-PAGE/Western analysis using Wnt1 antibodies. The amount of cell surface (S) Wnt1 in the immunoprecipitated fraction was normalized to the amount of Wnt1 in the total cell lysate (L; Fig. 3). Although our data indicate a possible trend with wild-type Wnt1 showing the highest levels of protein on the surface and Wnt1 C93A/S224A having the lowest levels, none of these differences are statistically significant (Student's t-test p = 0.11 for the comparison of Wnt1 and Wnt1 C93A/S224A). Nonetheless, our data indicate that it is unlikely that the mutation of the lipid modified residues prevents release of Wnt1 from the cell surface.

### C93 and S224 are required for clearance from the ER/Golgi

**Figure 4:**
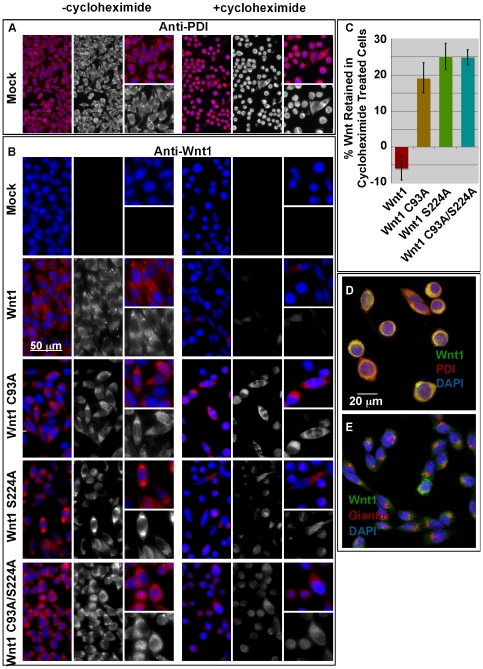
Mutation of the cysteine and/or serine causes an accumulation of Wnt protein in the endoplasmic reticulum. Stably transfected L cells were grown in the presence (+cycloheximide) and absence (-cycloheximide) for 14–21 hours. Cells were then fixed and stained for PDI (**Panel A**) or Wnt1 (**Panel B**). Images were collected with a SPOT camera attached to a Nikon E600 compound microscope. A scale bar is shown for reference. This experiment was carried out twice. **Panel C:** For quantitation of these studies, images were collected via confocal microscopy. Fields for imaging were selected while visualizing only the DAPI channel. NIH ImageJ was used to measure the cell intensities in the Wnt1 channel. The data shown represents the sum of 3 independent replicates with a total of 9 fields counted (3 for each replicate). At least 120 cells were counted for each data point. Error bars indicate +/− standard error. In **Panels D** and **E**, confocal analysis of Wnt1 C93A/S224A of cycloheximide treated cells is shown. Cells were immunostained with anti-Wnt1 (green), anti-PDI (red; **Panel D**), and anti-Giantin (red; **Panel E**). DAPI (blue) was used to visualize the nuclei.

To compare the passage of Wnt1 variants through the secretory pathway, we grew L cells stably transfected with wild-type or mutant Wnt1 in the absence or presence of cycloheximide, an inhibitor of translation. As a control, we also monitored the expression of Protein Disulfide Isomerase (PDI), a resident ER protein (Fig. 4A,B,D). As expected, PDI was detected at similar levels in the absence and presence of cylcoheximide in mock and Wnt1 transfected L cells (Fig. 4A,B; note that cycloheximide treated cells are somewhat rounded up). Our data show that wild-type Wnt1 is readily visible in the secretory pathway of untreated cells, but not detectable after cycloheximide treatment (Fig. 4B,C). By contrast, 19–25% of the single and double Wnt1 mutants were retained in the secretory pathway of cycloheximide-treated cells (Fig. 4B,C). As was the case with secretion, the differences in cell retention for wild-type and mutant versions of Wnt1 are highly significant (p<1×10^−5^).

To assess the localization of acylation deficient Wnt1 in cycloheximide treated cells, we used confocal microscopy to co-localize Wnt1 C93A/S224A with PDI or Giantin, a resident Golgi marker [30]. Our confocal images show extensive localization with PDI and only nominal co-localization with Giantin. Thus, the vast majority of retained Wnt protein is localized to the ER (Fig. 4D, E).

We conclude that Wnt1 lipid modifications to the cysteine and serine are required for proper transit through the secretory pathway. Thus, it is likely that the failure of lipid-deficient Wnt1 to successfully navigate the secretory pathway significantly contributes to the observed secretion defects.

### The lipid-modified cysteine and serine residues have distinct roles in the regulation of ß-catenin dependent signaling

**Figure 5:**
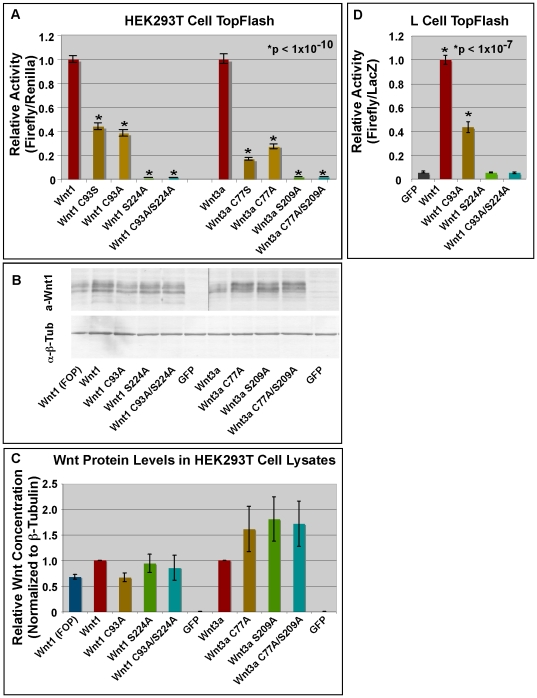
Wnt1 S224 is more critical than C93 for ß-catenin dependent signaling. **Panel A:** HEK293T cells were transfected with 8XSuperTop/FopFlash [64], renilla luciferase, and indicated pcDNA expression constructs. DNA concentrations were held constant within each series of experiments. Values shown reflect the experimental data (Firefly/Renilla) minus FopFlash (Firefly/Renilla). The relative luciferase activity of wild-type Wnt1/Wnt3a was set at 1. Single and double Wnt1/Wnt3a mutants show significantly less activity than wild-type Wnts (p<1×10^−10^). Differences between C93S and C93A mutants are not significant nor are those between S224A and C93A/S224A. Error bars indicate standard error for at least 15 data points from 3 independent replicates. **Panel B:** HEK293T cells were transfected as in Panel A, lysed, subjected to SDS-PAGE, and blotted onto PVDF. Blots were probed with anti-Wnt1 or anti-ß-tubulin. **Panel C:** The Western blots were scanned and analyzed using NIH ImageJ. This experiment was performed 3 times. Error bars represent +/− standard error. **Panel D:** LS/L cells were then transiently transfected with constructs encoding GFP or wild-type/mutant Wnt1. As before, the relative luciferase activity of wild-type Wnt1 was set at 1. Wnt1 and Wnt1C93A show statistically significant increases in reporter activity as compared to GFP (p<1×10^−7^) while Wnt1S224A and Wnt1C93AS224A do not. Error bars represent standard error from at least 3 independent replicates with a total of 12 data points.

We then tested the functional roles of the cysteine and serine residues in regulating signaling via the ß-catenin dependent pathway. To do this, we assessed the ability of wild-type and mutant Wnt1/3a proteins to activate the SuperTopFlash reporter in HEK293T and LS/L cells. We first transiently transfected HEK293T cells with wild-type or mutant Wnt expression constructs as well as SuperTopFlash reporter constructs [31]. In contrast to what we have observed in L cells, our preliminary data shows that mutation of the cysteine and/or the serine residue has little or no effect on secretion from HEK293T cells (data not shown). Although Wnt1 (C93A) and Wnt3a (C77A) retained significant biological activity in HEK293T cells, it was reduced by 62 and 73% as compared to their wild-type counterparts (Fig. 5A; p<10^−10^). Wnt1 (S224A), Wnt1 (C93AS224A), Wnt3a (S209A), and Wnt3a (C77AS209A) showed no detectable biological activity (Fig. 5A). To ensure that the transiently transfected HEK293T cells produced equivalent amounts of wild-type and mutant Wnt1/3a protein, we performed Western Blots of transfected cell lysates with antibodies against Wnt1/3a and ß-tubulin (Fig. 1B,C). Quantitation of these data show that the levels of wild-type and mutant Wnt1 and Wnt3a (normalized to ß-tubulin) are indeed comparable. These results are fully consistent with those of Willert et al [14] who showed that Wnt3a C77A expressed in HEK293 cells retained significant, but not full, biological activity, despite being secreted as well as wild-type Wnt3a. Together, these data suggest that the observed differences in biological activity for Wnt1 C93A and Wnt1 S224A in HEK293T cells cannot be attributed to differential production or secretion.

We then extended these studies to transiently transfected LS/L cells (LS/L cells are L cells that are stably transfected with SuperTopFlash and ß-galactosidase) [31], [32]. In contrast to HEK293T cells, wild-type Wnt1 (Fig. 2) and Wnt3a proteins [29] are relatively efficiently secreted from L cells while lipid-deficient Wnts (such as Wnt3a S209A) show greatly reduced secretion (see Fig. 2) [13], [29]. Measurement of Luciferase activity in LS/L cells transiently transfected with wild-type and mutant Wnt1 constructs yielded results nearly identical to those obtained in HEK293T cells (Fig. 5D). Specifically, they show that although both the cysteine and the serine residues are required for normal signaling, the requirement for the serine residue is more critical than the cysteine in ß-catenin dependent signaling. Furthermore, because our studies show that the single and double mutants show equal levels of secretion (as per Fig. 2), these results indicate that the differences in biological activity observed for Wnt1C93A and Wnt1S224A in L cells are not the result of differential secretion.

### Wnt1 and Wnt3a stimulate L cell elongation via a ß-catenin independent pathway

**Figure 6:**
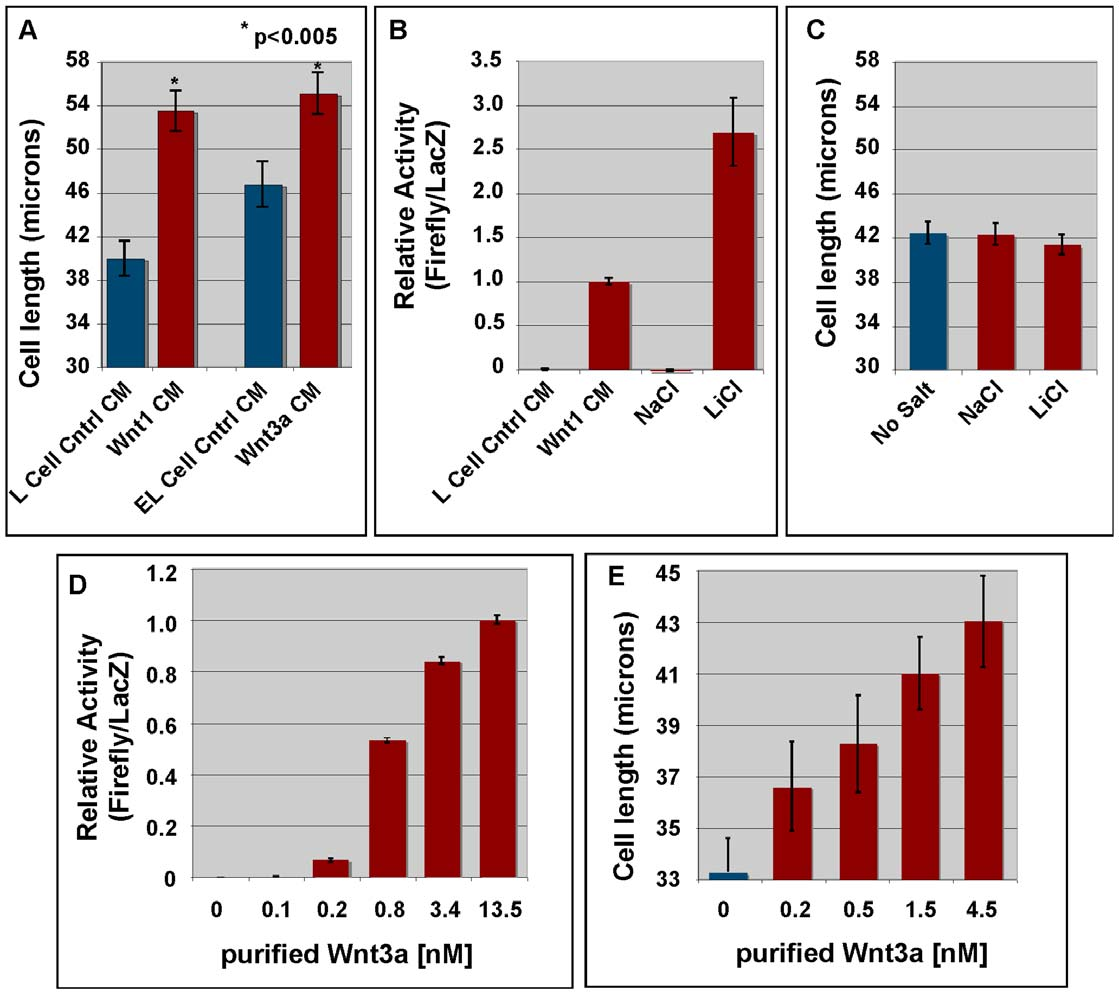
Wnt1/3a promote L cell elongation via a ß-catenin independent pathway. **Panel A:** Mock-transfected L cells were treated with control-conditioned media (Control-CM) or Wnt1/3a-conditioned media for 24 hrs. Note that Wnt1-CM was produced by standard L cells while Wnt3a was produced by E-cadherin transfected L cells (EL cells). Cells were then fixed, immunostained for PDI, and imaged with a confocal microscope. The length of the long axis of cells was measured using Adobe Photoshop (version 9.0.2). Error bars represent standard error. This experiment was repeated twice. At least 100 cells in 5 fields were measured. A Student's t-test shows that the difference in cell length between cells treated with control-CM and Wnt1- or Wnt3a-CM is statistically significant (p<0.005). **Panel B:** LS/L cells were treated with control-CM from mock-transfected L cells, Wnt1 conditioned medium, 33 mM NaCl, or 33 mM LiCl for 20–30 hrs and then assayed for activation of the SuperTopFlash reporter. **Panel C:** L cells were treated with 33 mM NaCl or LiCl for 24 hrs and then analyzed for cell elongation as in Panel A. Error bars represent the standard error from over 150 cells in two independent experiments. Neither NaCl nor LiCl has any significant effect on the length of the cells. **Panel D:** Purified mWnt3a was added to LS/L cells 24 hrs prior to conducting a dual luciferase assay to measure the activation of the SuperTopFlash construct. Error bars represent the standard error from 2 independent replicates with a total of 8 data points. **Panel E:** Mock-transfected L cells were incubated with purified mWnt3a for 24 hrs. Cells were then fixed, immunostained with anti-PDI, and imaged via confocal microscopy. The length of the long axis of individual cells was measured in Adobe Photoshop. Error bars represent the standard error from 2 independent replicates with a minimum of 107 cells measured for each data point.

During the course of our studies, we noticed that Wnt1-transfected L cells appeared to extend filopodia and were more elongated than mock-transfected cells. Because this phenotype had not previously been reported, we further characterized this morphological change. To rule out the possibility that observed changes were caused by our random selection of particular transfected L cell clones for study (ie: a clonal effect), we treated mock transfected L cells with control conditioned media or Wnt1/Wnt3a-conditioned media. We then measured the long axis of cells immunostained with antibodies against PDI, an ER marker, and compared the average length of cells treated with control-conditioned media to those treated with Wnt-conditioned media. Our results show that both Wnt1- and Wnt3a-conditioned media increased the length of the cells as compared to control-conditioned media (p<0.005; Fig. 6A), suggesting that elongation observed with stably transfected cells is not the result of a clonal effect.

Wnt ligands are known to signal via both ß-catenin dependent and independent pathways [17], [18], [19], [21], [33], [34], [35], [36], [37]. While the ß-catenin dependent pathway is well characterized, the details of the various ß-catenin independent pathways, including the so-called planar cell polarity and calcium pathways, remain ill-defined. To test whether the elongation observed in our assay could be induced by ß-catenin dependent signaling, we treated cells with LiCl, a well-known activator of ß-catenin dependent signaling [38]. Although addition of 33 mM LiCl robustly induced the SuperTopFlash reporter (Fig. 6B), it had no effect on cell length (Fig. 6C). These data demonstrate that activation of ß-catenin dependent signaling is not sufficient to promote cell elongation and suggest that the elongation of L cells is mediated via a ß-catenin independent pathway. We are currently investigating whether stable transfection of activated ß-catenin can replicate Wnt1/3a activity in the cell elongation assay. We have also initiated studies using pharmacological inhibitors to identify the Wnt signaling pathway involved in L cell elongation. Though we do not yet know the molecular nature of this pathway, we do know that it is distinct from the ß-catenin dependent pathway measured in our SuperTopFlash assay in that it cannot be activated by the addition of LiCl.

To determine whether the observed elongation of L cells was a direct effect of Wnt signaling, we then tested the ability of purified Wnt3a to promote elongation of L cells (Fig. 6E). For comparison, we also tested the ability of purified Wnt3a to activate the SuperTopFlash reporter that measures ß-catenin dependent Wnt signaling (Fig. 6D) [31], [32]. Dose response curves for the two assays show that cell elongation and activation of the SuperTopFlash reporter require similar concentrations of Wnt3a (Fig. 6D, E). Collectively, these data indicate that Wnt ligands directly promote cell elongation via a pathway that it likely to be ß-catenin independent.

**Figure 7:**
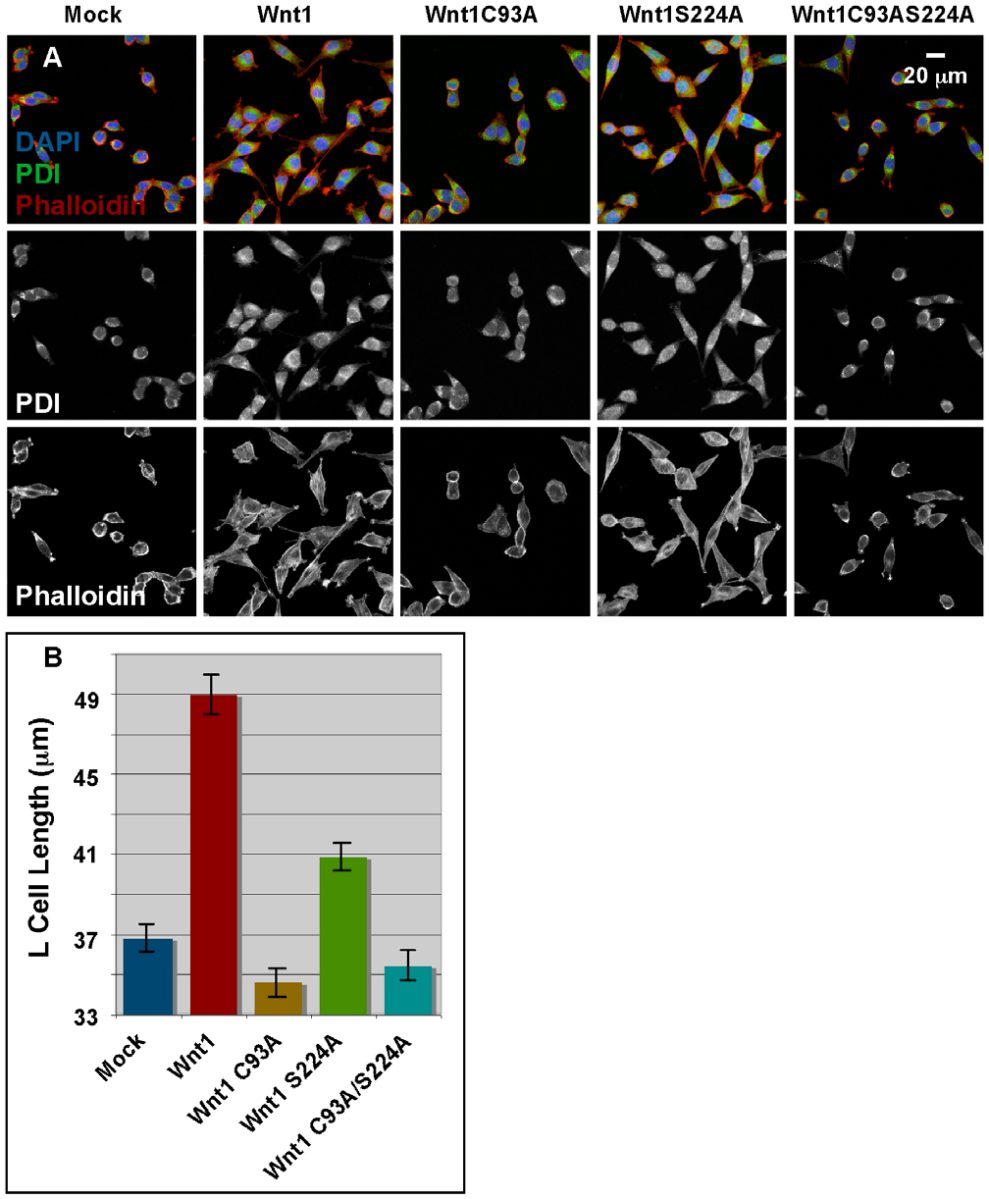
Wnt1 C93 is more critical than S224 for ß-catenin independent signaling in L cells. **Panel A:** L cells stably transfected with vector alone (mock), Wnt1, Wnt1C93A, Wnt1S224A, or Wnt1C93AS224A were stained with DAPI to visualize nuclei, anti-PDI to detect Protein Disulfide Isomerase and Phalloidin to detect Actin. Confocal images are shown (blue = DAPI; green = PDI; red = phalloidin). A scale bar is also shown for reference. Fluorescence in the DAPI channel was used to randomly select fields of view for imaging analysis. **Panel B:** The length of the long axis of PDI-stained cells was measured and graphed. A Student's t-test shows that the elongation of cells stably transfected with Wnt1 and Wnt1 S224A is statistically significant (p<1×10^−9^) as compared to mock-transfected cells while that of Wnt C93A and Wnt1 C93AS224A is not (p>0.3). 6 independent replicates with at least 21 total fields and 470 total cells were measured. Error bars show standard error.

To compare the roles of the lipid-modified cysteine and serine residues in β-catenin independent signaling, we assessed the morphology and length of mutant and wild-type L cells that were stained with anti-PDI and/or Phalloidin. Whereas the majority of mock-transfected cells were rotund or slightly elongated, Wnt1 transfected cells were substantially polarized/elongated with extensive protrusions (Fig. 7A). Consistent with these observations, measurement of the long axis of cells stained with anti-PDI showed that cells expressing Wnt1 were significantly longer than mock-transfected cells (Fig. 7A). Comparison of the effects of mutating the cysteine and/or serine residues further showed that mutation of the cysteine is far more deleterious to Wnt1 activity than mutation of the serine, which retained roughly half of the activity of wild-type Wnt1 (Fig. 7B). As in the SuperTopFlash reporter assay, doubly mutated Wnt1 had no detectable biological activity in the cell elongation assay. Nearly identical quantitative results were obtained for cells stained with Phalloidin (data not shown). These results suggest that the cysteine is more important than the serine in our ß-catenin independent elongation assay in L cells. Because we have already shown that the different L cells lines were producing similar amounts of Wnt protein (see t = 0, Fig. 2B) and the secretion of the mutants is similar, these data suggest that Wnt1 signaling via this unknown pathway requires the cysteine, but not the serine residue.

Cumulatively, these results demonstrate that the cysteine and serine residues are important regulators of biological activity. However, the relative contributions of these residues, and by inference, their respective lipid-modifications, are highly variant and depend on the signaling pathway assayed.

### Pharmacological inhibition of Porcn in L cells inhibits ß-catenin dependent, but not β-catenin independent signaling

**Figure 8:**
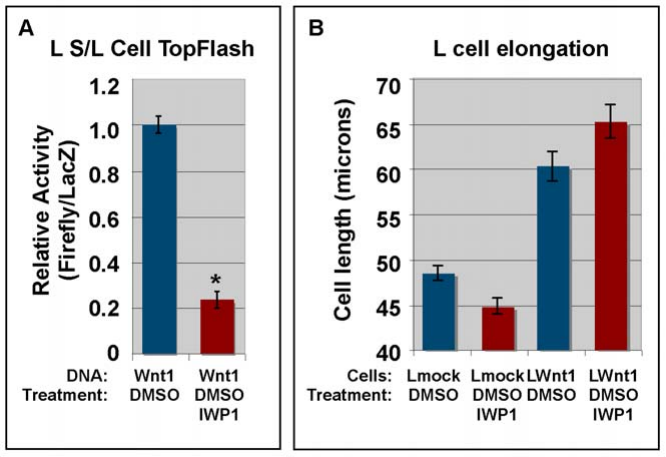
Pharmacological inhibition of Porcn inhibits ß-catenin dependent signaling in L cells. **Panel A:** L S/L cells were transiently transfected with Wnt1. 4 hrs post-transfection, the standard medium was changed to medium containing 500 nM IWP1 in DMSO or DMSO alone. Cells were incubated for 24 hrs and processed for the Dual light (Luciferase) assay. This assay shows that 500 nM IWP1 reduces ß-catenin dependent signaling by approximately 80% (p<1×10^−11^). Error bars show the standard error from 3 independent experiments with a total of 12 replicates. **Panel B:** Mock or Wnt1 stably transfected L cells were treated with DMSO (control) or IWP1 for 96 hrs. Cells were fixed, stained with DAPI/immunostained for PDI, and imaged via confocal microscopy (using the DAPI channel to randomly select fields). The long axis of the cells was measured in Adobe Photoshop (version 9.0.2). Error bars represent the standard error from 3 independent experiments with a minimum of 225 data points. A Student's t-test shows that IWP1 has no significant effect on cell length for either mock or Wnt1 transfected cells.

Porcn is an upstream regulator of Wnt lipid modifications. However, it is not known if it regulates the modification of both of the known acylated residues. To test the role of Porcn in β-catenin dependent and independent Wnt signaling in L cells, we transiently transfected LS/L reporter cells with Wnt1 and then treated with IWP1 [39], a pharmacological inhibitor of Porcn, prior to assaying for Luciferase activity. We performed a dose response curve for IWP1 (not shown) and found that 500 nM IWP1 was sufficient to reduce β-catenin dependent signaling by roughly 80% (Fig. 8A). To test the effect of IWP1 on our ß-catenin independent cell elongation assay, we treated mock or Wnt1 transfected L cells with DMSO (carrier) or 500 nM IWP1 (solubilized in DMSO) for 4 days (medium containing IWP1 was replenished after 48 hrs). Measurement of the long axis of PDI-stained cells shows that treatment of Wnt1 transfected cells with IWP1 had no significant effect as compared to DMSO (Fig. 8B; p>0.05). These results indicate that Porcn-dependent acylation of Wnt1 is primarily required for the ß-catenin dependent pathway in L cells.

### Porcn promotes the lipid modification of GFP-tagged Wnt1 peptides containing the conserved serine residue (S224), but not the conserved cysteine residue (C93)

**Figure 9:**
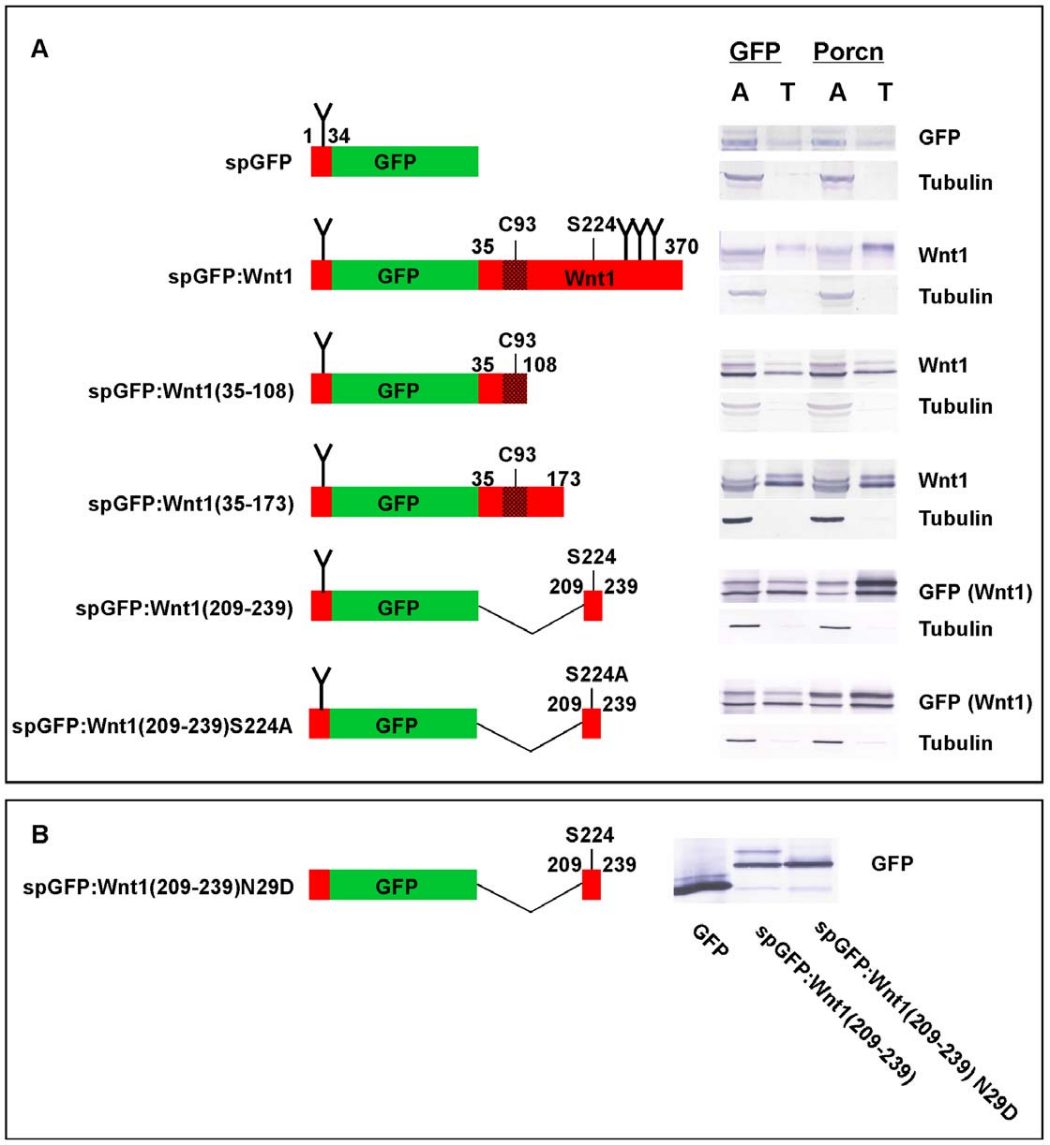
Porcn overexpression increases the hydrophobicity of spGFP∶Wnt1(209–239), but not spGFP∶Wnt1(35–108) in the TX-114 assay. **Panel A:** 293T cells were transfected with spGFP-tagged peptides in the presence of GFP (control) or mPorcD. A diagram of tested spGFP∶Wnt1 fusions is shown on the left. Wnt1 sequences are indicated in red while GFP sequences are indicated in green. Indicated numbers correspond to the residue position in full-length Wnt1. Consensus sites for the addition of N-linked carbohydrates are shown by a “Y”. The stippled region surrounding C93 indicates the antigen that was used to raise our monoclonal antibodies against Wnt1. Cells were incubated overnight, lysed, and extracted with TX-114 before analysis by SDS-PAGE/Western blot. spGFP∶Wnt1 fusions were detected with either Wnt1 or GFP antibodies. Blotting with ß-tubulin antibodies served as a control for the extraction as we have previously shown that ß-tubulin preferentially partitions to the aqueous phase. When co-transfected with GFP, all of the fusion proteins are found in both aqueous (A) and detergent/Triton X-114 (T) phases. The presence of ectopic Porcn promotes the partitioning of spGFP∶Wnt1, spGFP∶Wnt1 (209–239), and spGFP∶Wnt1 (209–239) S224A, but not spGFP∶Wnt1 (35–108) or spGFP∶Wnt1 (35–173) into the hydrophobic phase, indicating that 1) Porcn promotes the hydrophobic modification of a 31 residue peptide containing S224 2) that at least one additional site for the modification of Wnt by Porcn is likely to exist within the 31 residue peptide. These experiments were performed four times, on average. **Panel B:** A Western blot of cell lysates isolated from HEK293T cells transiently transfect with constructs encoding GFP, spGFP∶Wnt1(209–239), and spGFP∶wnt1(209–239) N29D is shown. The blot was probed with anti-GFP antibodies.

Our results demonstrating the involvement of Wnt1 S224 and Porcn in ß-catenin dependent signaling suggested the possibility that Porcn mediates the acylation of the serine residue, but not the cysteine residue. To test this prediction, we generated a series of GFP-tagged Wnt1 peptides spanning the cysteine and/or serine residues and then used a TX-114 phase separation assay to assess the ability of Porcn to promote the lipid-modification of these fusion proteins (Fig. 9). To ensure that peptide fusions were targeted to the secretory pathway, we appended residues 1–34 of Wnt1 to the N-terminus of *Aequorea* eGFP. These residues include a classic signal peptide (1–27) with the predicted cleavage site between residues 27 and 28 [40], [41]. Though the signal peptide is expected to be cleaved from the mature protein in the endoplasmic reticulum (ER), we refer to this protein as spGFP to distinguish it from cytosolic GFP. Seven additional residues (28–34) of Wnt1 were also included as they contain a consensus site for N-linked glycosylation, which is needed for efficient palmit(e)oylation of Wnt proteins [9]. Constructs encoding GFP-tagged Wnt1 fusion proteins were co-transfected into HEK293T cells along with cDNAs encoding cytosolic GFP (control) or Porcn. Cell lysates were separated into aqueous and detergent fractions by extraction with TX-114 and analyzed by Western blot. As controls, we show that Porcn does not increase the hydrophobicity of spGFP while it does promote the hydrophobicity of spGFP∶Wnt1 (full length). We then tested whether Porcn could cause an increase in the hydrophobicity of spGFP-tagged Wnt1 peptides spanning the two known acylated residues (C93 or S224). Porcn did not cause a shift of spGFP∶Wnt1(35–108) from the aqueous to the detergent phase (Fig. 9A). To test if additional residues might be required for Porcn activity, we also tested spGFP∶Wnt1(35–173). Once again, Porcn did not promote an increase in hydrophobicity of this fusion protein (Fig. 9A). By contrast, Porcn did increase the hydrophobicity of spGFP∶Wnt1(209–239), which contains sequences flanking and including S224.

Because each of the fusion proteins had at least one consensus site for the addition of N-linked glycosylation, we were not surprised to observe multiple bands in our TX-114 experiment. To ensure that the extra band observed for the constructs with just one glycosylation site was indeed caused by glycosylation, we compared the Western blot profile of a N29D mutation in the spGFP∶Wnt1 (209–239) to that of GFP and spGFP∶Wnt1(209–239) (Fig. 9B). As predicted, spGFP∶Wnt1(209–239) N29D migrated as a single band of the same size as the smaller band for spGFP∶Wnt1(209–239). Importantly, this band is larger than the GFP band, thus indicating that it is unlikely to be the product of proteolytic degradation. In sum, these data raise the possibility that the lipid-modification of C93 is Porcn-independent while that of S224 is Porcn-dependent.

To verify that S224 is indeed the site for the Porcn-dependent lipid modification, we also tested the ability of Porcn to promote a hydrophobic shift for spGFP∶Wnt1(209–239), in which serine 224 was replaced with an alanine (S224A) (Fig. 9A). Though this assay is highly qualitative, our data show that the percentage of protein shifted to the detergent phase by Porcn is greatly reduced for spGFP∶Wnt1(209–239) S224A as compared to spGFP∶Wnt1(209–239). Our data also indicate that Porcn still weakly promotes the hydrophobicity of spGFP∶Wnt1(209–239) S224A. These data are consistent with the Porcn-dependent modification of S224 and imply the existence of a secondary site for Porn-dependent modification.

**Figure 10:**
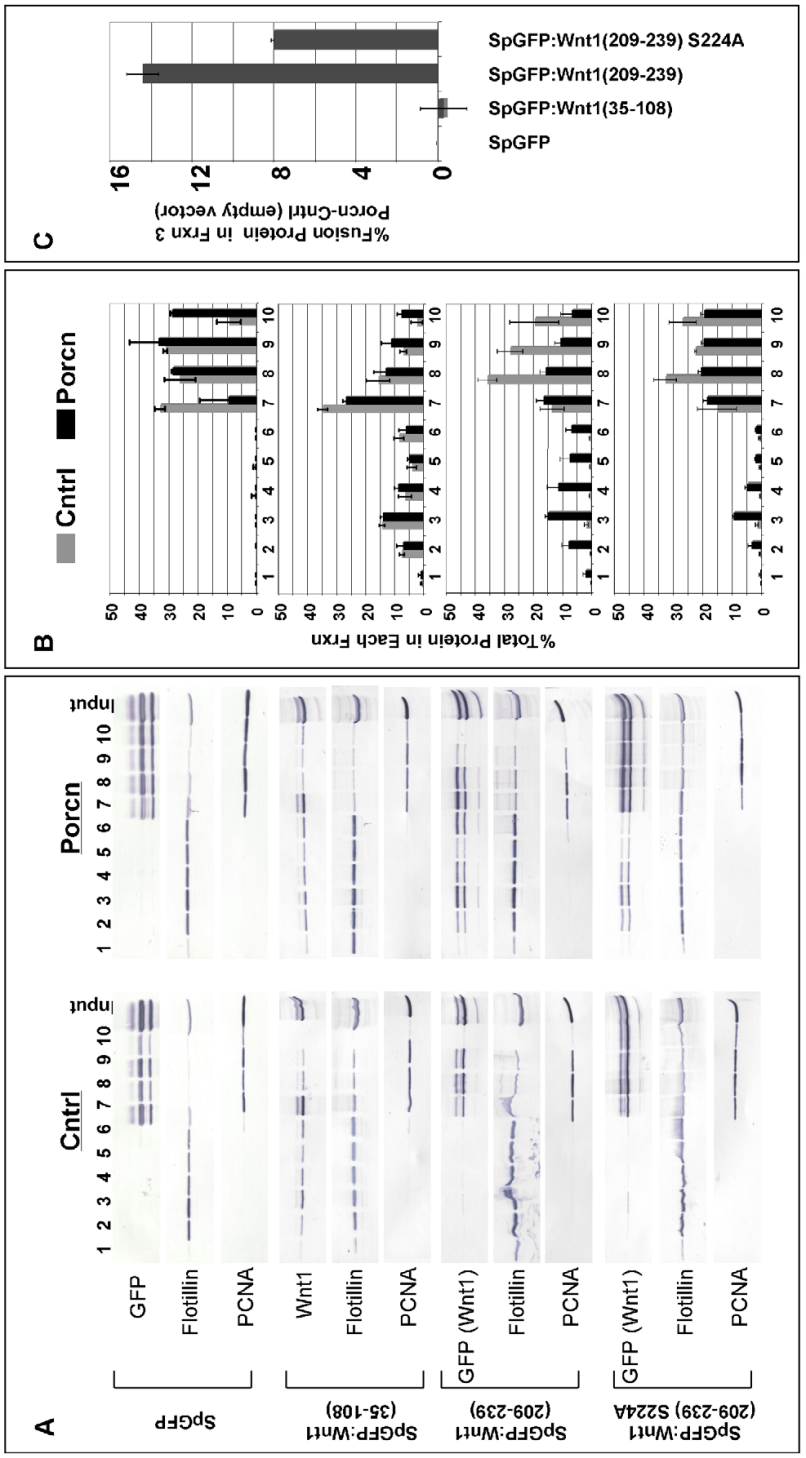
Porcn overexpression increases the association of spGFP∶Wnt1 (209–239), but not spGFP∶Wnt1 (35–108) with lipid rafts. HEK293T cells were transiently transfected with selected spGFP∶Wnt1 constructs along with empty vector (Cntrl) or Porcn. Cell lysates were separated by ultracentrifugation through an Optiprep density gradient. Fractions from the gradient were collected from top (1) to bottom (10). Western blots for Wnt1, Flotillin, and PCNA are shown in **Panel A**. Western blot data was then quantitated using NIH Image J. The graphical representations are shown in **Panel B**, where each bar corresponds to the percent of total protein in a particular fraction. The percentage of protein directed to fraction 3 (the primary lipid raft containing fraction) in a Porcn-dependent manner was calculated by subtracting the percentage of protein in Cntrl transfected cells from that in Porcn transfected cells (**Panel C**). The data in Panels B and C represent the average values obtained from 2–3 independent experiments. The co-transfection of cells with Porcn and SpGFP∶Wnt1 (35–108) had no effect on the levels of this fusion protein in lipid rafts. Co-transfection of cells with Porcn and spGFP∶Wnt1(209–239) or spGFP∶Wnt1(209–239) S224A did increase the relative proportion of fusion protein associated with lipid rafts.

We then sought to confirm our results using an independent assay designed to measure the ability of Porcn to target Wnt1 to lipid rafts. This assay is based on the demonstrated requirement for Porcn-dependent lipid modifications to localize Wingless (*Drosophila* Wnt1) to lipid rafts [15]. Though biological significance of this function is not yet known, this assay provided us with an important mechanism to confirm our results from the TX-114 assay. To assess the ability of Porcn to promote the association of spGFP∶Wnt1 peptides with lipid rafts, we co-transfected HEK293T cells with Wnt1 and either control (empty vector) or Porcn constructs. We used Western blot analysis to detect spGFP∶Wnt1 peptide in lipid rafts that were isolated by ultracentrifugation through a 5–40% Optiprep gradient. Controls for our density gradient separation include Flotillin-1, a protein enriched in lipid rafts, and PCNA, a nuclear protein. These controls reveal the presence of lipid rafts in fractions 2–5. Quantitation of these data showed that, as expected, overexpression of Porcn had no effect on the distribution of spGFP (Fig. 10). Consistent with the data from the TX-114 phase separation assay, our results indicate that overexpression of Porcn did not cause an increase in the relative proportion of spGFP∶Wnt1(35–108) that is localized to lipid rafts (Fig. 10). However, a significant proportion of the fusion was localized to lipid rafts in the absence of Porcn. Interestingly, this Porcn-independent localization of the spGFP∶Wnt1(35–108) fusion protein to lipid rafts is also independent of C93 as fusions bearing a C93A mutation were also localized to lipid rafts (data not shown). By contrast, overexpression of Porcn caused a significant shift in the distribution of spGFP∶Wnt1(209–239) into fractions containing lipid rafts (Fig. 10). Together, these data strengthen the notion that the palmitoylation of Wnt1 C93 is Porcn-independent while palmiteoylation of Wnt1 S224 is Porcn-dependent. Furthermore, replacement of S224 with an alanine quantitatively reduces the amount of spGFP∶Wnt1(209–239) directed to lipid rafts by Porcn by 44% (Fig. 10 B,C), thus confirming the importance of this residue in Porcn-dependent lipid modifications. The observation that Porcn can still promote the localization of spGFP∶Wnt1(209–239) S224A to lipid rafts is again consistent with the existence of at least one secondary site for Porcn in the 209–239 peptide.

To further test whether palmitoylation of Wnt1 C93/Wnt3a C77 is Porcn-independent, we designed one additional assay. For this assay, we wanted to rule out the possibility that the spGFP∶Wnt1(35–108) or spGFP∶Wnt1(35–173) fusion proteins lacked the proper sequences or folding needed for Porcn-dependent palmitoylation of C93/C77. To do this, we changed the cysteine residue to a serine residue. As our data show that Porcn is clearly able to promote the formation of oxyester linkages (to Wnt1 S224), we reasoned that if Porcn is also capable of promoting the lipid modification of the cysteine residue, it should still be able to modify Wnt1 or Wnt3a with a cysteine to serine substitution. As a consequence, we would expect C93S/C77S mutations to have less severe effects on biological activity than C93A/C77A mutations. To test this prediction, we compared the biological activity of Wnt1 C93S/C93A and Wnt3a C77S/C77A mutations in a TopFlash reporter assay carried out in HEK293T cells. Our data indicate that the reduction of the biological activity observed for Wnt1 C93S and Wnt3a C77S was highly significant as compared to wild-type Wnt1 and Wnt3a (p<10^−10^), but was strikingly similar to C93A and C77A mutants (p = 0.5; Fig. 5A). These data are consistent with the observation that only short peptides are generally required as substrates for palmitoylation reactions [42]. The most straightforward explanation is that Porcn does not promote the palmitoylation of C93 or C77 and that distinct enzymes are involved in the modification of cysteine and serine residues. Cumulatively, our findings show that Porcn-dependent modification of Wnt1 S224 preferentially regulates ß-catenin dependent signaling in vertebrate HEK293T and L cell lines.

## Discussion

The mechanisms by which palmit(e)oylation controls the activity and distribution of secreted proteins, such as Wnts, is poorly understood. In this manuscript, we systematically dissected the roles of conserved lipid-modified residues in the Wnt1 ligand in cultured HEK293T cells and L cells. In the course of these studies, we found that C93 and S224 have similar effects on the transit of Wnt through the secretory pathway and on Wnt secretion. However, comparison of the relative roles of C93 and S224 in activation of two distinct signaling pathways revealed a novel role in influencing the choice of signaling pathway used. Notably, the Porcn-dependent lipid modification of S224 specifically promotes signaling via the ß-catenin dependent pathway in L cells.

Our data showing an equal role for the lipid-modified cysteine and serine residues in the secretion of Wnt1 both agree and disagree with other reports. For example, our data showing that mutation of Wnt1 S224 leads to reduced secretion in L cells is entirely consistent with parallel data obtained for Wnt3a S209 in L cells [13]. At first glance, our data showing the requirement of the Wnt1 C93 for secretion appears to be at odds with the report from Willert et al, who showed little effect of a Wnt3a C77A mutation in HEK293 cells. However, our preliminary studies suggest that this apparent discrepancy reflects the differences in cell lines used (L cells vs HEK293 cells). By contrast, our data are not in accordance with those of Franch-Marro et al, who showed that HA-tagged Wingless required the cysteine, but not the serine, for secretion in *Drosophila* [43]. It is likely that these apparent discrepancies reflect 1) differences in the model systems that were utilized or 2) differences between the use of untagged (us) and tagged [43] Wnt proteins.

Though our data show a clear role for Wnt S- and O-palmit(e)oylation in secretion, they add to the growing body of evidence that contradicts the conventional wisdom that the sole function of Wnt palmit(e)oylation is to promote/permit secretion [10], [14], [44]. First, although our studies show that Wnt1 mutants bearing single or double mutations are secreted at similar levels, the biological activities of these mutants vary significantly. Second, L cells treated with IWP1, a specific inhibitor of Porcn, exhibit reduced signaling in a ß-catenin dependent assay, but not our ß-catenin independent cell elongation assay (despite the intrinsically equal levels of Wnt secretion in the two assays). Third, we have obtained preliminary data in HEK293T cells confirming that although the secretion of Wnt1 C93A and S224A mutants closely mirrors that of wild-type Wnt1 (data not shown), their biological activity in a SuperTopFlash assay is significantly reduced (Fig. 4) [7].

Though our data showing that mutation of C93 and S224 reduce secretion in L cells are consistent with a model in which knockdown/mutation of Porcn cause the retention of Wnt protein inside cells [13], [45], [46], the mechanism by which overexpression of Porcn in HEK293T cells causes an increase in the cell-associated Wnt protein remains unresolved [7]. Because palmitoylation of proteins has previously been shown to regulate protein stability [3], [4], we had suspected that overexpression of Porcn would cause an increase in Wnt stability. However, the finding that the presence or absence of the lipid-modified cysteine and serine residues does not significantly affect Wnt1 protein stability is not consistent with this prediction. Recently, however, a paper from Gao et al showed that only a small fraction of Wnt3a produced by stably transfected L cells is actually palmitoylated [47]. Thus, it is likely that the vast majority of our wild-type Wnt1 was, in fact, not palmit(e)oylated. These results lead us to believe that differences in stability might have been missed our pulse chase assay.

Our data provide the basis for a working model that describes the role of Wnt1 lipid modifications in regulating the activation of distinct pathways. In this model, Wnt1 ligands with dual modifications to C93 and S224 are able to signal via multiple pathways. By contrast, Wnt1 ligands bearing single modifications to either the cysteine or serine residue preferentially promote signaling via a single pathway. In L cells, for instance, Wnt1 that is palmiteoylated on S224 preferentially signals via the ß-catenin pathway while Wnt1 that is palmitoylated on C93 primarily signals via a ß-catenin independent pathway. Though we do not know the underlying mechanism for this observation, we speculate that differentially palmit(e)oylated Wnt1 preferentially activates distinct cell surface receptors and thus, signaling cascades.

A number of Wnt ligands are known to signal through multiple pathways. However, the vast majority of data to date suggest that Wnt1 and Wnt3a signal almost exclusively via the ß-catenin dependent pathway. Thus, our data showing the activation of a ß-catenin independent signaling pathway by Wnt1 and Wnt3a in L cells represents an important finding. The molecular characterization of this pathway is still in progress.

Our studies strongly suggest that distinct enzymes mediate the palmit(e)oylation of Wnt1 C93 and S224. Specifically, our data is most consistent with a model in which Porcn promotes palmiteoylation of S224, but not the palmitoylation of C93. Our data are consistent with those of Ching et al, who showed that DWntD, a *Drosophila* Wnt ligand that possesses the conserved cysteine residue, but lacks the conserved serine residue (and a short span of flanking residues), functions in a Wls and Porcn-independent manner [16]. Our data are also in full alignment with those from Coombs et al, who showed that the interaction of Wls with Wnt3a required S209 and Porcn, but not C77 [26]. Nonetheless, we fully appreciate that biochemical studies with purified Porcn will be required to unambiguously show that it has a direct role in the palmiteoylation of S224 and not the palmitoylation of C93.

These data lead to the interesting observation that related members of the MBOAT superfamily preferentially catalyze the formation of distinct types of linkages. Hhat, for example, promotes N-linked acylation of the N-terminal cysteine residue [48]. Though it is not known whether the amide linked palmitate results from the direct formation of the amide bond or by an intramolecular S-N shift of the palmitate from the sulfhydryl group to the terminal amino group, substitution of the cysteine with a serine shows that Hhat is unable to catalyze the formation of oxyester linkages [48]. By contrast, Ghrelin acyl transferase and Porcn promote O-linked acylation [49], [50]. The mechanisms by which these related MBOATs catalyze the formation of distinct linkages will be an interesting avenue of inquiry. In addition, the identity of the enzyme that catalyzes the addition of palmitate to the cysteine residue also remains to be determined. Our data suggest that this enzyme will catalyze the formation of thioester, but not oxyester linkages.

We also showed data suggesting the existence of a secondary site(s) for the modification of vertebrate Wnts by Porcn. Though our results were somewhat unexpected, our data are easily reconciled with those of Takada et al and Doubravska et al, who showed that Wnt3a S209A did not incorporate any ^3^H-palmitate in their assays [13], [44]. In their experiments, Wnt3a was overexpressed while Porcn was not. Thus, it is possible that the level of endogenous Porcn was not sufficient to permit detection of secondary palmit(e)oylation sites. In our experiments, both Wnt and Porcn were overexpressed thus, increasing the chances of observing palmit(e)oylation of secondary (higher Km) sites.

Our data also serve to resolve some contradictory data about the role of palmit(e)oylation in targeting Wnts to lipid rafts. In 2004, Zhai et al noted that Porcn-dependent lipid modifications were required for targeting Wingless to lipid rafts [15]. Recently, Doubravska et al found that Wnt1 (S224A) was still targeted to lipid rafts and therefore, concluded that lipid modifications were not required for lipid raft targeting. The presence of at least one additional Porcn-dependent lipid modification site could easily explain this result. Further, our results showing that overexpression of Porcn increases the amount of a GFP∶Wnt1 fusion protein spanning the serine residue that is targeted to lipid rafts argues that Porcn (and by inference, Wnt1 lipid modifications) does indeed promote the targeting of Wnt1 to lipid rafts.

We have shown that Porcn-dependent lipid modifications to the serine residue preferentially control Wnt1 signaling via the ß-catenin dependent pathway in L cells. These data are consistent with recent reports from the Rossant and Murtaugh labs, in which Porcn deficient mice die during gastrulation and have defects in ß-catenin dependent signaling [51], [52]. Data from the Korinek lab showing a primary requirement for the serine residue in ß-catenin dependent signaling in *Xenopus* is also consistent with our results [44]. Though it would be tempting to speculate that Porcn-dependent modifications to the serine residue are universally required for ß-catenin dependent signaling, data from several labs do not support this notion. For instance, in *Drosophila*, mutation of C93 in HA-Wg causes a greater reduction in ß-catenin dependent signaling than mutation of S239. Likewise, in zebrafish, knockdown of Porcn using morpholinos causes no overt reduction in ß-catenin dependent signaling in developing embryos (Dr. Shinji Takada, personal communication). Similarly, although we have also acquired evidence that Porcn-dependent modifications are not required for at least one ß-catenin independent pathway in L cells, it seems unlikely that all ß-catenin independent pathways function independently of Porcn. For instance, in *C. elegans*, Porcn (Mom-1) is required for signaling in the Wnt asymmetry pathway, which is distinct from the “canonical” ß-catenin dependent pathway [53], [54], [55]. Likewise, in zebrafish, knockdown of Porcn causes defects in convergence and extension, which are normally associated with defects in the planar cell polarity branch of ß-catenin independent signaling pathways (Dr. Shinji Takada, personal communication). Because the availability of cell surface receptors plays a significant role in dictating which signaling pathway is used by a Wnt ligand [32], [33], it is possible that tissue specific differences in the expression of cell surface receptors may yield variable effects.

The palmit(e)oylation of Wnt proteins by distinct proteins further suggests the interesting possibility that the lipid modification of each site could be independently regulated. Because Porcn is expressed in a nearly ubiquitous pattern [52], regulation of Porcn would likely be at the post-translational level. Further experiments are required to understand the regulation of the enzymes that palmit(e)oylate Wnt proteins.

Our data have important ramifications for the treatment of Wnt-driven cancers and Focal Dermal Hypoplasia, a human syndrome caused by mutations in Porcn [56], [57]. Future studies will be required to determine which signaling pathways are regulated by Porcn-dependent Wnt lipid modifications in affected cells.

## Materials and Methods

We like to thank Dr. Lawrence Lum for his generous contribution of IWP1; Dr. Randall Moon (University of Washington) for supplying Super8xTopflash and Super8xFopflash [31]; Dr. Roel Nusse for providing L cells, L cells transfected with SuperTopFlash and ß-galactosidase, EL and EL-Wnt3a cells, as well as the pPGK and pPMPIneo vectors. Dr. Andrew McMahon (Harvard University) for chick Wnt1 partial cDNA [58], mouse Wnt1 and mouse Wnt3a cDNAs [59], [60]; Dr. Elena Frolova (Washington University) for partial cWnt1 cDNA [61]; Dr. Tatsuhiko Kadowaki (Nagoya University) for mPorcD cDNA [62].

### Reagents

Other materials include: TX-114 (Fisher); Lipofectamine 2000, Phalloidin-Alexa Fluor 633 (Invitrogen); Fugene HD (Promega); Mouse anti-GFP JL-8 (Clontech); FITC or Cy3 conjugated goat anti-rabbit IgG (H+L), Cy3 conjugated goat anti-mouse IgG IgM (H+L) and goat anti-mouse IgG (H+L)-AP, anti-rabbit DyL 649 (Jackson Laboratories); Dual Luciferase Reporter (Promega); anti-ß-tubulin, anti-PCNA (Santa Cruz Biotechnology); Dual Light System (Applied Biosystems); HEK293T cells (Developmental Studies Hybridoma Bank); anti-HA (Upstate); anti-Flotillin (BD Biosciences); anti-PDI (Calbiochem); anti-Giantin (Abcam); Optiprep (Accurate Chemicals); Tran^35^S label (MP Biomedicals); Protein A/G agarose beads, Sulfo-NHS-LC-biotin, NeutraAvidin Agarose (Pierce); En^3^Hance (Perkin Elmer); purified mouse Wnt3a (R&D Systems).

### Plasmids

Full length cDNAs for chick Wnt1 and chick Wnt3a have not been isolated. Thus, we used a construct with the mWnt3a signal peptide fused to cWnt3a coding sequence [63] and mWnt1 signal peptide fused to cWnt1 coding sequence [7]. We used overlapping extension to generate point mutations in cWnt1 (C93 or S224) and cWnt3a (C77 or S209) to change the residues to alanine or serine.

For uniformity of expression in cell culture studies, the following cDNAs were cloned into pcDNA3.1(-)A (Invitrogen): cWnt1, cWnt1 C93A, cWnt1 C93S, cWnt1 S224A, cWnt1 C93A S224A, cWnt3a, cWnt3a C77A, cWnt3a C77S, cWnt3a S209A, cWnt3a C77A S209A, spGFP, spGFP∶Wnt1, spGFP∶Wnt1(35–108), spGFP∶Wnt1(35–173), spGFP∶Wnt1(209–239), spGFP∶Wnt1(209–239)S224A, spGFP∶Wnt1(209–239)N29D, mPorcD, and eGFP. The HA epitope tag was also appended to the C-terminus of the Wnt1 and Wnt1 C93A S224A constructs. The myc/his tag encoded by sequences in pcDNA3.1 was intentionally excluded from all constructs. For stable transfections the following cDNAs were cloned into pPGK: cWnt1, cWnt1 C93A, cWnt1 S224a and cWnt1 C93A S224A.

### Antibodies

Generation of monoclonal antibodies against cWnt1 and cWnt3a is described in Galli et al 2007 [7]. For cWnt1 antibodies, clone 5F1-G11-D1 was used for immunostaining and western blots, while clone 7B3-A10-F9 was used for immunoprecipitations. For the Wnt3a antibodies, clone 3E9-1B11-H3 was used for all studies.

### Immunoprecipitations

HEK293T cells were transfected in a 6 well plate with 3.3ug of eGFP, cWnt1-HA, or cWnt1 C93A S224A-HA (all in pcDNA 3.1) using the FUGENE HD transfection kit according to the recommended protocol. After 24–26 hrs incubation, the cells were washed 1× with PBS, lysed with 1 ml of TENT buffer (20 mM Tris, pH 8, 150 mM NaCl, 2 mM EDTA, 1% TX-100 containing leupeptin, aprotinin, and PMSF) and incubated at 4°C with rocking for 30 minutes. After incubating the lysates at −80°C for at least 1 hr, lysates were thawed and cleared by centrifugation at 15,800*×g* for 15 minutes at 4°C. The cleared lysates was divided into 2 tubes and immunoprecipitated with anti-Wnt-1 (MAb clone 7B3-A10-F9) or anti-HA antibodies. Initial lysates and immunoprecipitated samples were run on duplicate gels and probed for Wnt1 (using MAb clone 5F1-G11-D1) or HA. This experiment was performed twice; images were analyzed using NIH Image J.

### L cell transfections

Unless noted otherwise, all cells were grown in standard medium (DMEM containing 10% fetal bovine serum, 4 mM L-glutamine, and 1× penicillin/streptomycin). Stably transfected L cells were co-transfected with 8 ug of pPGK.cWnt1, pPGK.cWnt1C93A, pPGK.cwnt1S224A or pPGK.cwnt1C93A S224A along with 0.8 ug of pPMPI.neo on 60 mm plates with Lipofectamine 2000. After 24 hours the medium was changed to include 800 ug/ml of G418. The medium was changed every 2–4 days for three weeks with fresh G418. G418 resistant cells were clonally diluted and grown for 2 weeks. Conditioned media from the wells that contained just one clone were collected and subjected to Western blot analysis with anti-Wnt1 MAb. Positive clones were grown and re-screened. The following clones were selected and used: cWnt1 2H5; cWnt1C93A 1A5; cWnt1S224A 2A8 and cWnt1C93A S224A 2C11.

### Pulse chase studies

L cells stably transfected with Wnt1, Wnt1C93A, Wnt1S224A or Wnt1C93A S224A were split onto 35 mm plates in standard medium one day prior to pulsing. On the day of the pulse, the standard medium was replaced with DMEM without cysteine or methionine for 15 minutes and then changed to 0.7 ml of the same medium containing 0.25 mCi/ml of tran^35^S label. After a 4-hour pulse, the medium was replaced with 1 ml of standard medium. Media and cell lysates were collected at designated time points. A portion of the cell lysate was subjected to Western blot analysis with ß-tubulin as a protein loading control. The remaining lysates and media were immunoprecipitated using anti-Wnt1 monoclonal antibodies along with protein A/G agarose beads. Upon completion of the immunoprecipitation, the beads were resuspended in 1.5× sample loading buffer, boiled and subjected to electrophoresis on a 12.5% acrylamide gel. The gel was incubated for 1 hour with En^3^Hance, incubated for 30 minutes in ice cold 0.5% glycerol in water and dried between cellophane. Dried gels were analyzed by STORM Phosphorimager.

### Detection of Wnt1 on the cell surface

L cells stably transfected with Wnt1, Wnt1C93A, Wnt1S224A or Wnt1C93A S224A were plated to 6 well plate 1 day prior to labeling. The next day, cells were incubated with media containing 100 ug/ml of Heparin for 4 hours (to release Wnts bound to HSPGs). Cells were then washed 3 times with ice cold PBS (pH 8) before adding 0.75 ml of 3 mM Sulfo-NHS-LC-biotin in PBS pH 8. The plate was put on ice and incubated at 4°C for 15 minutes. The reaction was stopped by washing cells (3×) with PBS (pH 8) containing 100 mM glycine. The cells were then lysed by the addition of 1 ml of ice cold TENT containing 0.1%SDS, rocked at 4°C for 30 minutes, triterated with 25G needle, and frozen at −80°C for at least 1 hour. After thawing, the lysates were cleared by centrifugation at 15,800*×g*, 4°C for 15 minutes. Cleared lysates were precipitated with NeutrAvidin Agarose beads in TENT containing 0.1%SDS overnight at 4°C. After washing the beads were then washed (3×) with TENT containing 0.1% SDS, biotin-labeled proteins were released by boiling in SDS-PAGE sample buffer. Cleared lysates and immunoprecipitated proteins were separated by SDS-PAGE and Wnt1 was visualized by Western blot. Blots were analyzed using NIH Image J. This experiment was replicated 6 times.

### Cycloheximide Treatment

L cells stably transfected with Wnt1, Wnt1C93A, Wnt1S224A and Wnt1C93A S224A were seeded onto 8 well glass slides and incubated overnight in standard medium. The following day, fresh medium was added in the presence or absence of 10 ug/ml of cycloheximide. Cells were incubated for 14–21 hrs, fixed and immunostained with various combinations of anti-Wnt1, anti-PDI, and anti-Giantin antibodies. Following incubation with fluorophore-coupled secondary antibodies and DAPI, cells were imaged with a SPOT camera attached to a Nikon E600 or a Zeiss LSM 710 confocal laser scanning microscope. Identical exposures are shown for all images. These experiments were performed 2–3 times.

To quantitate the data obtained from cycloheximide treated cells, cells were imaged using a Zeiss LSM 710 confocal laser scanning microscope using the same laser power and gain for all images for the Wnt1 channel utilizing the 63× oil objective. Fields for imaging were selected while visualizing only the DAPI channel. To measure cell intensities, only the Wnt channel was opened into Image J. After outlining the cells using the free hand selection tool, the average intensity was measured and recorded. Data includes three independent experiments with 3 fields measured each time. The total number of cells analyzed for each data point ranged from 122–167 cells.

### Super 8X TopFlash reporter assay

HEK293T cells were plated onto 24-well plates one day prior to transfection to yield density of 90–100% at transfection. Cells were transfected with Lipofectamine 2000. DNA quantities used in transfections are as follows: pcDNA3.1 GFP, pcDNA3.1 cWnt1, pcDNA3.1 cWnt1C93A, pcDNA3.1 cWnt1C93S, pcDNA3.1 cWnt1S224A, pcDNA3.1 cWnt1 C93A S224A, pcDNA3.1 cWnt3a, pcDNA3.1 cWnt3a C77A, pcDNA3.1 cWnt3a C77S, pcDNA3.1 cWnt3a S209A, cWnt3a C77A S209A at 0.25 ug/well; Super8xTopFlash or Super8xFopFlash at 0.01 ug/well; RL-CMV at 0.01 ng/well. Cells were incubated overnight, lysed and measured as per Promega Dual-Luciferase Reporter Assay protocol. Luciferase measurements were carried out in a TD-20/20 luminometer.

### Dual Light Assay

L S/L reporter cells (L cells stably transfected with SuperTop and ß-galactosidase) were seeded at 100,000 cells/well in a 96 well plate 1 day prior to transfection or media exchange. L S/L cells were transfected with 0.1 ug GFP or Wnt1 wildtype, single or double mutants with Fugene HD as per protocol (Promega). For experiments with IWP1, medium containing either 0.01% DMSO or 500 nM IWP1 in 0.01% DMSO was added 4 hrs post-transfection. For experiments containing purified mWnt3a or LiCl, the standard medium was exchanged with medium containing either pure mWnt3a at the indicated concentrations (holding BSA constant at 0.0013%) or 33 mM LiCl or NaCl. Cells were incubated for 20–30 hrs, lysed and measured as per Dual Light System protocol. Measurements were taken with a Berthold Microlumat Plus LB 96 V luminometer.

For Western blots, HEK293T cells were transfected as per above. The following day, cells were lysed and subjected to SDS-PAGE/Western blot analysis. Blots were probed with Wnt1 or ß-tubulin antibodies. This was carried out 3 times. Blots analyzed using NIH Image J.

### Cell elongation assay

For experiments using Wnt1/3a conditioned medium, L cells stably transfected with empty vector (mock-transfected) were seeded at 30,000 cells/well in a 8 chamber glass slide. Three to four hours later the medium was changed to 1/2 conditioned medium (either control, Wnt1 or Wnt3a) diluted in standard medium and incubated for approximately 24 hours. For the experiments with LiCl, mock-transfected L cells were seeded at 20,000–30,000 cells/well in a 8 chamber glass slide. The cells were allowed to adhere for 3 to 4 hours to overnight. The standard medium was replaced with medium containing 33 mM NaCL or LiCL and treated for approximately 24 hours. For experiments with purifed mouse Wnt-3a, mock-transfected L cells were seeded at 5,000 to 7,500 cells/well in 8 chamber glass slides and allowed to adhere for 3 to 4 hours. The standard medium was changed to medium containing pure mWnt3a at the indicated concentrations holding BSA at 0.0013%. The cells were subsequently incubated for 24 hours prior to analysis. For experiments using stably transfected L cells, cells transfected with empty vector, wild-type, or mutant Wnt1 constructs were seeded at 30,000 cells/well in a 8 chamber glass slide and allowed to adhere overnight. For IWP1 experiments, stably transfected mock and Wnt1 expressing L cells were seeded at 3,500 to 5,000 cells/well in 8 chamber glass slides and allowed to adhere for 3 to 4 hours. After replacing the standard medium with medium containing 500 nM of IWP1 (or DMSO as a control), cells were incubated for 48 hrs prior to replacing the medium with fresh medium (and IWP/DMSO). Cells were the incubated for an additional 48 hrs to yield a total incubation time of 96 hrs. Cells were then fixed and probed with anti-PDI and/or Phalloidin and/or anti-Wnt1. Fields for analysis were selected in the DAPI channel and images were collected on a Zeiss LSM 710 confocal laser scanning microscope with a 40× oil objective. Measurements were collected using Adobe Photoshop version 9.0.2.

### TX-114 Separation Assay

This assay was carried out as per Galli et al, 2007 [7]. After extraction, samples were precipitated, resuspended, and boiled in 1xSDS-PAGE sample buffer prior to electrophoresis and electroblotting. Western blots were probed with anti-Wnt1, anti-Wnt3a, anti-GFP and/or anti-ß-tubulin. Alkaline phosphatase-conjugated secondary antibodies were used for detection.

### Optiprep Density Gradient

HEK293T cells were plated onto 6 well plates. For each construct, 4 ug/well was transfected with Lipofectamine 2000 and the plates were incubated overnight. The cells were washed 1× with PBS and then 400 ul of ice cold 100 mM Tris pH 7.5 containing 150 mM NaCl, 2 mM EGTA, 0.5 mM PMSF, 1% TX-100, 10 ug/ml leupeptin, and 5 ug/ml aprotinin was added. The cells were triterated and then transferred to a microcentrifuge tube. The lysates were centrifuged at 5000×*g* for 5 minutes a 4°C. Optiprep (60% iodixanol in water) was added to 200 ul of cleared lysate to a final concentration of 40% Optiprep. A 40% (bottom) −5% (top) Optiprep gradient was generated by overlaying 400 ul of the lysates (in 40% Optiprep) with 1.2 ml 30% Optiprep and 400 ul 5% Optiprep. Samples were centrifuged at 100,000×*g* for 5 hours at 4°C. Fractions of 200 ul were collected, precipitated, and analyzed by Western blot. Western blots were probed with anti-Wnt1, anti-GFP, anti-flotillin and anti-PCNA. Alkaline phosphatase-conjugated secondary antibodies were used for detection.
